# The impact of dose on the real-world effectiveness of vortioxetine in outpatients with mdd in greece

**DOI:** 10.1192/j.eurpsy.2021.905

**Published:** 2021-08-13

**Authors:** E. Papalexi, P. Kakkavas, D. Vassos, T. Mylonaki, D. Partsafyllidis, S. Mageiria, C. Nestoris, A. Galanopoulos, A. Ettrup

**Affiliations:** 1 Medical, Lundbeck Hellas, Athens, Greece; 2 Psychiatric, Private Office, Athens, Greece; 3 Psychiatric, Private Office, Thesalonik, Greece; 4 Psychiatric, Private Office, Thesaloniki, Greece; 5 Medical, H Lundbeck A/S, Valby, Denmark

**Keywords:** Non-interventional, Vortioxetine, Depression, Dose

## Abstract

**Introduction:**

The current treatment goal in Major Depressive Disorder (MDD) is functional recovery (Zimmerman M et al, 2012). However, finding the “right dose for the right patient” may be challenging and the dose-response relationship for antidepressant efficacy remains controversial (Hieronymus F et al, 2016). Efficacy evaluated by MADRS increases with higher vortioxetine doses, based on meta-analysis data (Thase ME et al, 2016).

**Objectives:**

The aim of this exploratory analysis was to assess the impact of different doses on vortioxetine effectiveness in clinical practice in Greece.

**Methods:**

In this non-interventional study, open-label vortioxetine was administered at a flexible dosage (5-20 mg/d). Patients receiving 5/10 mg vortioxetine (group A), at the end of the study, were compared to patients receiving 15/20mg vortioxetine (group B). At baseline, 1 and 3 months, depressive symptoms and functioning were assessed by MADRS and SDS. Multiple regression was used for the statistical analyses.

**Results:**

The study included 336 MDD patients. At the end of the study, 64.3% (n=200) of patients were receiving 15/20 mg vortioxetine. Higher vortioxetine dose at month 3 was significantly correlated with higher MADRS total score at baseline (p<0.001). SDS total score change from baseline to month 3 was significantly associated with vortioxetine dose (p<0.001), with group A and group B showing improvements of -9.2±8.2 and -12.1±6.0, respectively- whereas such association was not observed for MADRS total score.

**Conclusions:**

In conclusion, patients with more severe depressive symptoms were treated with higher antidepressant doses. However, beyond symptom improvement, vortioxetine effectiveness on patient functioning seems to increase with higher doses.

**Conflict of interest:**

A. Galanopoulos and E. Papalexi are full-time employees in Lundbeck Hellas. A. Ettrup is a full-time employee in H. Lundbeck A/S.

